# An American Text-Book of Applied Therapeutics

**Published:** 1897-06

**Authors:** 


**An American Text-Book of Applied Therapeutics.** Edited by J. C. WILSON, M.D., assisted by AUGUSTUS A. ESHNER, M.D. Two Volumes. Pp. 1326. London: The Rebman Publishing Co. (Ltd.) Philadelphia: W. B. SAUNDERS. 1896.

The contents of these volumes are an important contribution to therapeutics, based upon the application of the results of investigation to the uses of the practising physician. Of the forty-two contributors all are American except Laveran, who writes the section on malarial fever, and the late Dr. Beaven Rake, to whom was assigned the responsibility of dealing with leprosy. Notwithstanding the statement in the preface that the work would be issued in one volume, it comes to us in two imposing volumes consecutively paged, an apparent afterthought which is certainly a concession to convenience. The articles composing this work are by no means *farrago libelli*, which forms so large a portion of many text-books on therapeutics, but every statement is made upon a sure scientific basis, not upon empiricism or mere credulity. The separate sections amount to nearly eighty in number, and therefore can not receive a detailed criticism within the space at our disposal; at the same time we must venture to record that Dr. J. T. Whittaker, of Cincinnati, in the instructive article on tuberculosis, strenuously recommends the tuberculin treatment. Having passed in review the many remedies proposed for tubercle of recent years, he says: "Thus every day sees born a new remedy; a few months pass, and the remedy falls into oblivion." He strongly believes of tuberculin, that "in the dose of 0.00005 gram the remedy is entirely free from danger, and employed in the proper cases, after the method described, is the most valuable of all the single methods of treatment of incipient tuberculosis." As may be expected, Dr. Whittaker approves also of hygienic, climatic and dietetic—especially alcohol, which he prefers to cod-liver oil—modes of treatment in co-operation with tuberculin.

The work is by no means narrowly restricted to the consideration of therapeutics. Etiology and pathology are incidentally considered in an equally scientific spirit, although at much less length. A few excellent illustrations are included, such as those in the articles on diphtheria and diseases of the mind.

The printing and paper are very good, and we have not discovered a single uncorrected typographical error. No doubt some time must have now elapsed since some of the articles were written; occasionally they suggest a deficiency of that "new thing" which, like the ancient Athenian, the modern therapeutist desires to see and hear. But taken altogether we wish to express our sincere admiration and respect for this unique work.

